# Human Temperaments. VIII.—The Practical Man


**Published:** 1916-08-05

**Authors:** 


### HUMAN TEMPERAMENTS.

### VIII.—THE PRACTICAL MAN.*

The practical man is, as his title implies, concerned with doing things; and in as far as he recognises that action is the most important function of animate beings, he has grasped a great truth; but what he does not appreciate so well is that action, if it is to be successful, must be based upon knowledge, and he is apt to despise knowledge. He has a great contempt for the theorist, whom he calls a theoretical fool, and he does not appreciate that as all action is founded on knowledge, so all knowledge is supposititious; nor does he appreciate that, much as he despises theory and the theorist, he is himself the most thoroughgoing theorist of them all. His favourite expression is "The fact is" so-and-so, that which he takes for a fact being usually a wild and groundless speculation. "The fact is," the practical doctor tells his hypochondriac patient, "your liver is out of order, and I shall give you some medicine to stimulate its action." The supposition that the patient's symptoms are due to derangement of the functions of the liver is a wild speculation, resting on no basis of fact, and on no evidence whatever. The doctor has a very hazy notion of what the functions of the liver are, and if he were asked to connect these functions with the patient's symptoms, he could not do so. To do him justice, he would not try. He would regard the question as "theoretical trash," and if it were pushed, he would probably say, "How do I know? Because I am a practical man. I give him some medicine to stimulate his liver, and you will see that will cure him." Accordingly, he administers taraxacum and podophyllin, or calomel and jalap, and his patient does recover. "There!" says he triumphantly, "Now who was right?" He was right in supposing that taraxacum and podophyllin or c. cum j. would cure his patient, but there is not the slightest evidence that any of these drugs could or did stimulate the liver; nor has he any inkling of what would happen if the functions of the liver were stimulated. The supposition that they acted on the liver is as wild and baseless a speculation as the supposition that the functions of the liver were disordered. But, it may be said, What does it matter as long as the patient recovers? And it is true that as long as the patient recovers it does not matter—to the patient. But it matters a great deal to the doctor. In this case his speculation did no harm; but this case is exceptional. He did not, in fact, base his action on his hypothesis, though no doubt he thought he did. The real reason for the administration of the drugs was neither the belief that the liver was disordered nor the belief that the drugs would rectify this disorder, though no doubt both these beliefs existed. The real reason for his action was that the same drugs had cured a similar disorder in other cases; and whether they cured the disorder by acting on the liver or in other ways did not matter in the least. In this case no harm was done; but the harm that has been done, and is daily being done, by the practical man in proceeding on a false hypothesis which he does not know to be an hypothesis, but fondly believes is an established fact, is incalculable. The number of patients who have been killed by attempts to "disperse the peccant humour" or to "bring on the crisis," is no doubt very great, but it is to be doubted whether these attempts had more disastrous effects than attempts to purify the blood, or to reduce the acidity, or to correct an alimentary toxæmia, or to reduce the intake of purin.

### Words and Things.

The practical man reads little. He is unable to grasp an abstraction. His vocabulary is limited; but he makes up for the limitation of his knowledge of words by his implicit belief in the power of the words that he does possess. He does not distinguish between words and things: a word is to him as good as a thing, and a verbal explanation which explains nothing is to him completely satisfactory. He is puzzled by the behaviour of his pears, which turn black and fall off the tree as soon as they have reached the size of a crab or a sloe; but tell him it is a blight, and he is satisfied, and does not care to inquire further. His rubber syringe, which was so elastic and pliable, is become hard and rigid, and when it is squeezed it cracks and splits. What has happened to it? Tell the practical man that it is perished, and he is quite satisfied. He knows now as much as he cares to know, and thinks he knows more than he knew before he was in possession of the explanatory word. He sees that criminality is not prevented by punishing criminals, and declares that the only true remedy is education; but if you can get at his notion of education it consists in teaching reading and writing, and a few other things, including Latin grammar; and even the practical man would not consider a knowledge of Latin grammar an effectual antidote to criminality. But in using the term "education" as a preventative of criminality, he does not attach any meaning to education. He does not go behind the word, and "education" is to him as effectual a word as "blight" or "perished." The word alone is sufficient. The passion for giving names to things has been inherent in the human race ever since Adam gave names to all the animals, and no doubt it is convenient in speaking of things to have names to speak of them by; but the practical man is apt to regard the name of a thing as not only an inherent and inseparable quality of the thing, but as a key to all its other qualities; hence to him a name is satisfying. He is not so very far removed from the savage who believes that knowledge of his enemy's name gives him power over that enemy.

* The previous articles appeared on May 6, 13, 27, June 10 and 24, and July 8 and 22, 1916; and a letter on the subject on May 20.

Hence all the diagnostic efforts of the doctor who is a practical man are devoted to finding a name for his patient's malady, and when he has attached a name to it, he is content. He now takes it for granted that he knows all about the disease there is to be known. To picture to himself the train of disordered processes in their sequence is an effort that it never occurs to him to make. Like the savage, he regards possession of the name as possession of power over the thing named.

The Obvious Obscures the Important.

The practical man sees what is obvious, and misses what is important if it is not obvious also. The practical men laughed at Copernicus and Galileo, for they *saw* the sun rise and set, and surely seeing is believing! Practical men clamour for taxes to be laid on foreign-made goods, so that the money shall go to enrich the British producer and not the foreigner. The payment to the foreigner for his goods is obvious, and cannot be overlooked; what is overlooked is that this payment is made, not in money, but in goods, and that these goods must be produced by the British producer, to whom, and not to the foreigner, the money goes directly; and another thing that is overlooked is that the British producer does not part with his goods to the foreigner as a gift, nor even upon equal terms, but that he parts with what he does not want in exchange for what he does. He parts with what he can produce in exchange for what he cannot, or for what he can produce easily, and therefore cheaply, in exchange for what he can produce only with difficulty, and therefore expensively. Practical men clamour for the State to do this and that or to find the funds for doing this and that, with the implied conviction that the State holds a Fortunatus' purse, and that what the State does is pure gain to everyone, and costs nothing to anyone but the State. That the State has no money except what it takes out of the pockets of its citizens is not sufficiently obvious to impress them.

The practical man takes short views. He adapts direct means to immediate ends. If the progress of the horse up the hill is arrested, the remedy of the practical man is not to take some of the bricks out of the cart, but to flog the horse. If the patient is emaciated, exhausted, and hungry, the remedy of the practical man is to give him a good square meal. That his unfortunate victim has turned the corner of typhoid fever, and that the good square meal is likely to be fatal, is unknown to him. Rags and dirt are signs of poverty, and poverty is to be relieved by charitable gifts, and therefore the benevolent practical man is profuse in almsgiving to the ragged and dirty, and so accentuates poverty instead of relieving it.

Practicality Less Crude.

The practical man is less crudely practical than he was. He has discovered by many an experience and many an example that the longest way round is sometimes the shortest way there. He has a glimmering of knowledge that after all the most obvious remedy is not always the best remedy; that diarrhœa is sometimes best treated, not with astringents and opiates, but with castor oil; that the true remedy for poverty is not to give alms but to give opportunity; that the result of manuring his fruit-trees is more likely to be increase of wood than increase of fruit; that the remedy for discontent is not repression and coercion but the removal of its causes; that, in short, action, to be efficient, must be founded upon knowledge; and therefore he craves for knowledge. But his impulsion to immediate action is so strong that he is ready to seize upon anything that purports to be knowledge, and to act upon it. He wants to be doing something to remedy the ills of which he has a keen appreciation, and he has neither the patience to wait for a rational remedy nor usually the intelligence to judge whether a remedy is rational or not; so he plunges. While detesting "the theoretical fool," he does not recognise that, whatever his action, it must be founded on supposition, and as he cannot distinguish supposition from fact, he is at the mercy of every crank who puts forward a supposition and calls it a fact. He used to believe that gout was due to excessive drinking of port wine; he now knows that it is due to excessive production of uric acid, and that this again is due to consuming too much purin. He used to believe that poverty could be relieved by almsgiving; he now knows that the proper way to relieve poverty is by the formation of committees and the extension of the suffrage. He used to believe that shirking and cowardice were vices to be punished; he now knows that they are manifestations of a laudable tenderness of conscience. The practical man must be doing something. He is not content to wait, and watch, and investigate, until he knows the right thing to do. He must do something, even if it is the wrong thing, which it very often is. It is for this reason that we always order two tablespoonsful three times a day. It is doing something; and as the majority of our patients are practical persons, they demand that something shall be done. The illness runs its course, and most illnesses run their natural course towards recovery. In these our two tablespoonsful seldom do good, and let us hope they seldom do harm, to the patient. Their real function is not to affect the patient, but to satisfy the urgent need of his practical female relatives to be doing something to help him.

